# Antiviral and Neuroprotective Role of Octaguanidinium Dendrimer-Conjugated Morpholino Oligomers in Japanese Encephalitis

**DOI:** 10.1371/journal.pntd.0000892

**Published:** 2010-11-23

**Authors:** Arshed Nazmi, Kallol Dutta, Anirban Basu

**Affiliations:** National Brain Research Centre, Manesar, Haryana, India; Centre for Cellular and Molecular Biology (CCMB), India

## Abstract

**Background:**

Japanese encephalitis (JE), caused by a mosquito-borne flavivirus, is endemic to the entire south-east Asian and adjoining regions. Currently no therapeutic interventions are available for JE, thereby making it one of the most dreaded encephalitides in the world. An effective way to counter the virus would be to inhibit viral replication by using anti-sense molecules directed against the viral genome. Octaguanidinium dendrimer-conjugated Morpholino (or Vivo-Morpholino) are uncharged anti-sense oligomers that can enter cells of living organisms by endocytosis and subsequently escape from endosomes into the cytosol/nuclear compartment of cells. We hypothesize that Vivo-Morpholinos generated against specific regions of 3′ or 5′ untranslated regions of JEV genome, when administered in an experimental model of JE, will have significant antiviral and neuroprotective effect.

**Methodology/Principal Findings:**

Mice were infected with JEV (GP78 strain) followed by intraperitoneal administration of Morpholinos (5 mg/kg body weight) daily for up to five treatments. Survivability of the animals was monitored for 15 days (or until death) following which they were sacrificed and their brains were processed either for immunohistochemical staining or protein extraction. Plaque assay and immunoblot analysis performed from brain homogenates showed reduced viral load and viral protein expression, resulting in greater survival of infected animals. Neuroprotective effect was observed by thionin staining of brain sections. Cytokine bead array showed reduction in the levels of proinflammatory cytokines in brain following Morpholino treatment, which were elevated after infection. This corresponded to reduced microglial activation in brain. Oxidative stress was reduced and certain stress-related signaling molecules were found to be positively modulated following Morpholino treatment. *In vitro* studies also showed that there was decrease in infective viral particle production following Morpholino treatment.

**Conclusions/Significance:**

Administration of Vivo-Morpholino effectively resulted in increased survival of animals and neuroprotection in a murine model of JE. Hence, these oligomers represent a potential antiviral agent that merits further evaluation.

## Author Summary

Japanese encephalitis (JE) is caused by a flavivirus that is transmitted to humans by mosquitoes belonging to the *Culex sp*. The threat of JE looms over a vast geographical realm, encompassing approximately 10 billion people. The disease is feared because currently there are no specific antiviral drugs available. There have been reports where other investigators have shown that agents that block viral replication can be used as effective therapeutic countermeasures. Vivo-Morpholinos (MOs) are synthetically produced analogs of DNA or RNA that can be modified to bind with specific targeted regions in a genome. In this study the authors propose that in an animal model of JE, MOs specifically designed to bind with specific region of JE virus (JEV) genome, blocks virus production in cells of living organisms. This results in reduced mortality of infected animals. As the major target of JEV is the nerve cells, analysis of brain of experimental animals, post treatment with MOs, showed neuroprotection. Studies in cultured cells were also supportive of the antiviral role of the MOs. The potent anti-sense effect in animals and lack of obvious toxicity at the effective dosage make these MOs good research reagents with future therapeutic applications in JE.

## Introduction

The genus *Flavivirus* is composed of more than 70 different closely related species [1]. Many flaviviruses are arthropod-borne and causes significant human diseases. Among these, the four serotypes of dengue virus (DENV), yellow fever virus (YFV), West Nile virus (WNV) and Japanese encephalitis virus (JEV) are categorized as emerging global pathogens [2]. JEV is a mosquito-borne, positive sense, single stranded RNA virus, responsible for frequent epidemics of encephalitis, predominantly in children, in most parts of Southeast Asia and adjoining regions. It is the causal factor for 30,000–50,000 cases of encephalitis occurring every year and accounts for about 10,000 deaths annually with serious neurological squeal in the survivors [3]. JEV has been expanding its ‘geographical footprint’ into previously non-endemic regions and with several billion people at risk, Japanese encephalitis (JE) represents an internationally emerging concern in tropical and sub-tropical countries. Currently three types of JE vaccine are in use- the inactivated mouse-brain derived, the inactivated cell-culture derived and the live attenuated cell-culture derived. However, there are limitations for their usage in terms of availability, cost and safety [4]. At present, chemotherapy against JEV is largely supportive and not targeted towards the virus. A lot of avenues has been explored in the past and are also being currently tried, so as to develop a safe and effective molecule that would be able to prevent the virus from replicating within the host.

The JEV genome is approximately 11 kb in length that carries a single long open reading frame (ORF) flanked by a 95-neucleotide 5′ untranslated region (5′ UTR) and a 585-neucleotide 3′ UTR. The ORF encodes a polyprotein which is processed by viral and cellular proteases into three structural and seven non structural proteins [5], [6]. The 5′ and 3′ UTRs of the JEV genome contain conserved sequence elements and can form conserved stem loop structure. 5′ UTR contain secondary structures which are required for the formation of translation pre-initiation complex [7]. JEV requires long range RNA-RNA interaction between 5′ and 3′ regions of its genome for efficient replication; one such interaction occurs between a pair of 10 complementary nucleotides, located in coding sequence for the capsid protein at 136–146 nucleotides from 5′ end of the genome, and 3′ cyclization sequence, commonly denoted as 3′CSI (3′ conserved sequence I) located at 104–114 nucleotides from 3′ end of the genome [8], [9]. The 3′CSI is highly conserved across members of JEV serocomplex, indicating the possibility that RNA elements within the 5′ and 3′ UTRs in JEV genome are essential for its replication.

Anti-sense oligonucleotides have been shown to be effectively used as therapeutic agents against viral infection. In one such study siRNA generated against the cd loop-coding sequence in domain II of the viral Envelope protein (which is highly conserved among all flaviviruses because of its essential role in membrane fusion) has been found to protect against lethal encephalitis [10]. Similarly siRNAs has also been generated against various nonstructural proteins of JEV and were found to be effective in inhibiting viral replication [11], [12]. Anti-sense approach has also been employed to inhibit flaviviral replication by generating anti-sense molecules against RNA elements within the 5′ and 3′ UTRs in flaviviral genome. In one such approach, DNAzyme against 3′ UTR of JEV genome has be found to be effective in controlling virus infection in a murine model [13]. Under the same approach but with different kind of anti-sense oligonuleuotide called Morpholino, flaviviral replication has been inhibited in cultured cells as well as in animal models [14], [15].

Morpholino oligomers are single stranded DNA analogues containing same nitrogenous bases as DNA but joined by backbone consisting of morpholine rings and phosphorodiamidate linkages [16]. For efficient delivery into cells these Morpholino are often conjugated with arginine rich peptide [17]. However, in the current study we have used a different type of Morpholino oligomer called Vivo-Morpholino against 3′CSI and one of the secondary structures present in 5′ UTR of the JEV genome. Vivo-Morpholino are specialized type of non-peptide Morpholino oligomers, conjugated with a new transport structure that provides effective delivery into a wide variety of tissues in living animals, thereby raising the possibilities of their use as therapeutic agents. The transporter comprises of a dendritic structure assembled around a triazine core which serves to position eight guanidinium head groups in a conformation effective to penetrate cell membranes. Vivo-Morpholinos have also been shown to effectively enter and function within cultured cells [18]. Vivo-Morpholinos are also cost effective, non immunogenic, and stable under physiological conditions as compared to other types of Morpholinos. This study was designed to evaluate whether the use of Vivo-Morpholinos as therapeutic agents, is possible in an experimental model of JE. We intend to show that these specifically designed Vivo-Morpholinos are effective in countering the viral load in the body, thereby imparting significant protection to the animals that were infected with a lethal dose of JEV.

## Materials and Methods

### Ethics statement

All animal experiments were approved by the institutional animal ethical review board named “Institutional Animal and Ethics Committee of National Brain Research Centre”. The animal experiment protocol approval no. is NBRC/IAEC/2007/36. Animals were handled in strict accordance with good animal practice as defined by Committee for the Purpose of Control and Supervision of Experiments on Animals (CPCSEA), Ministry of Environment and Forestry, Government of India.

### Virus and cells

Vero cells (a kind gift from Dr. Guruprasad Medigeshi, Translational Health Science and Technology Institute, Gurgaon, India) and Neuro2A (obtained from National Centre for Cell Science, Pune, India) cells were grown in DMEM (Dulbecco's modified Eagles medium, supplemented with 10% fetal bovine serum (FBS) and antibiotics. The GP78 strain of JEV was propagated in suckling BALB/c mice and their brains were harvested when symptoms of sickness were observed. A 10% tissue suspension was made in MEM (minimum essential medium), followed by centrifugation at 10,000× g and finally filtered through a 0.22 µ sterile filter [19]. JEV was titrated by plaque formation on Vero cell monolayer. Vero cells were seeded in six-well plates to form semi-confluent monolayer in about 18 h. Cell monolayer were inoculated with 10-fold serial dilutions of virus samples made in MEM containing 1% FBS and incubated for 1 h at 37°C with occasional shaking. The inoculum was removed by aspiration and the monolayers were overlaid with MEM containing 4% FBS, 1% low-melting-point agarose and a cocktail of antibiotic–antimycotic solution (Gibco, Invitrogen Corporation, Grassland, NY, USA) containing penicillin, streptomycin, and amphotericin B. Plates were incubated at 37°C for 72–96 h until plaques became visible. To allow counting of the plaques, the cell monolayer was stained with crystal violet after fixing the cells with 10% formaldehyde.

### Morpholino oligonucleotides

**Figure 1:**
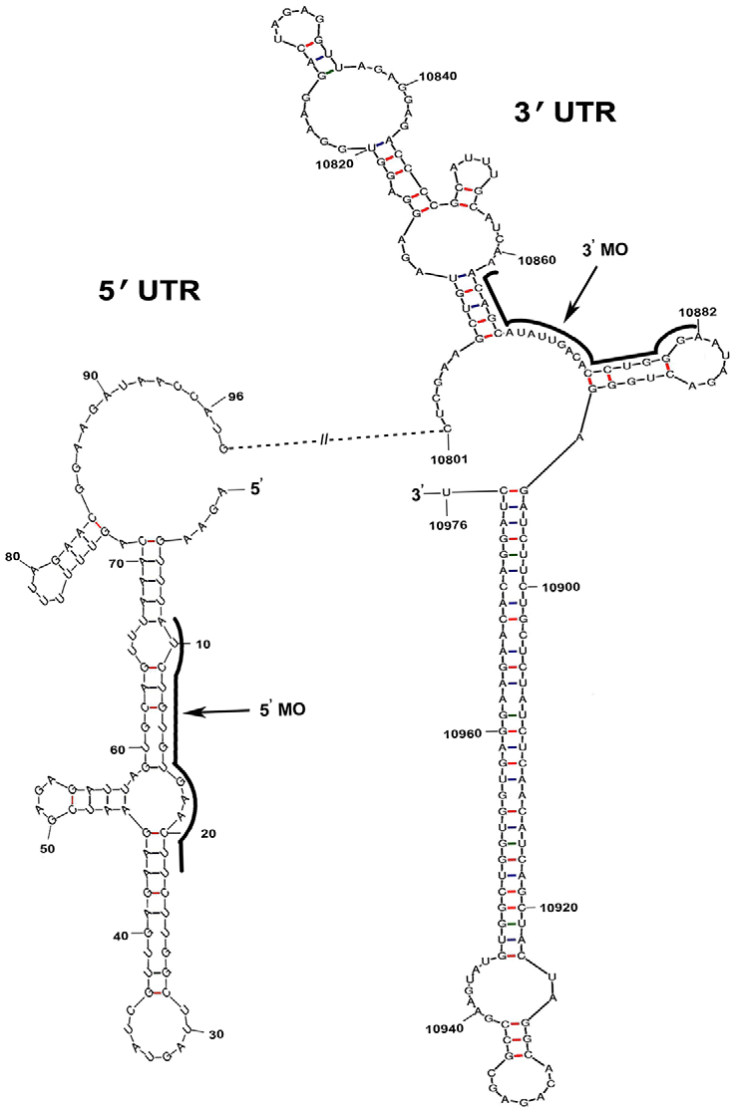
Regions of the JEV (GP78 strain) genome that are blocked by the MOs. Schematic diagram showing the secondary structure of the 3′ and 5′ UTR of JEV genomic RNA as predicted by the Mfold program [41]. The regions blocked by the 3′ MO and 5′ MO are marked by black curved lines and marked with arrows. Numbers in the smaller font refer to the nucleotide position of the JEV genome.

All Vivo-Morpholino (MO) oligos were commercially procured from Gene Tools LLC, (Philomath, OR, USA). MOs were designed to be complementary to sequences in the JEV (GP78 strain) genome, as shown in *Table 1*. These oligonucleotides targeted specific regions in the 3′ and 5′ UTRs of the JEV genomic RNA *(* *Figure 1* *)*. A 21 base scrambled MO of random sequence (SC-MO) was used as a negative control in all the experiments. All MO sequences were screened with BLAST (http://www.ncbi.nlm.nih.gov/BLAST) against primate and murine mRNA sequences and the SC-MO was additionally screened against all flaviviral sequences. All MOs were procured in 300 nanomole quantities as a liquid of 0.5 mM stock (approximately 4 mg/mL) in buffered saline. They were diluted with sterile 1× PBS to achieve desired concentrations, and stored at 4°C as aliquots.

**Table 1:** Names, sequences and target locations of used Vivo-Morpholinos.

Morpholino name	Morpholino sequence(5′-3′)	Targeted nucleotides in the JEV genome(GenBank: AF075723.1)
3′ MO	TCC CAG GTG TCA ATA TGC TCT T	10861–10882
5′ MO	AAG CCA AGA AGT TCA CAC AGA T	9–22
SC-MO	ACT CCA TCG TTC AGC CTC TGA	NA

NA- Not applicable.

### Animal model and treatment schedule

Five to six weeks old BALB/c mice of either sex were randomly distributed into 5 groups- Sham, JEV-infected, JEV-infected and treated with scrambled Morpholino (JEV+SC-MO), JEV-infected and treated with Morpholino against viral 3′ conserved region (JEV+3′ MO) and JEV-infected and treated with Morpholino against secondary structure in the 5′UTR of viral RNA (JEV+5′ MO). Initially each group contained 8 animals. Animals belonging to all groups except Sham were infected with 3×10^5^ plaque forming units (PFU) of JEV (GP78 strain) and that day was considered as day zero [20]. Animals of Sham group received equal volume of filtered MEM. Starting from 3 h post infection on day zero, 100 µg of SC-MO, 3′ MO and 5′ MO, diluted in 0.1 mL of sterile 1× PBS (corresponding to 5 mg/kg body weight), were administered to animals belonging to JEV+SC-MO, JEV+3′ MO and JEV+5′ MO groups respectively, once per day, for 5 consecutive days. Animals belonging to the Sham-treatment group received equal volumes of sterile 1× PBS only. Survivality of animals in each group following JEV infection and Morpholino treatment were monitored daily upto 15 days post JEV infection (or till their death, whichever was earlier). Toxicity of the Morpholinos in mice was evaluated by weight loss and abnormal behavioral & clinical observations (including tremors, ruffled fur, hunching, ataxia, gait abnormalities), in a masked manner to minimize bias [14], [20].

### Cytokine bead array

Mouse cytokine bead array (CBA) kits were used to quantitatively measure cytokine levels in mouse whole-brain lysates. 50 µL of bead mix containing a population of beads with distinct fluorescence intensities that have been coated with capture antibodies for different cytokines, and 50 µL of whole-brain lysates were incubated together, along with equal volume of phycoerythrin (PE)-conjugated detection antibodies, for 2 h at room temperature, in dark. The beads were then washed and re-suspended in 300 µL of supplied 1× wash buffer. The beads were acquired using Cell Quest Pro Software in FACS Calibur and analyzed using BD CBA software (Becton Dickinson, San Diego, CA). Standard curve was prepared by incubating 50 µL of supplied mouse inflammation standards with 50 µL of bead mix and PE-conjugated detection antibodies [21].

### Immunoblotting

Protein concentrations of whole brain lysates were estimated by Bradford method. Sample volumes containing 20 µg of protein were electrophoresed on polyacrylamide gel and transferred onto nitrocellulose membrane. After blocking with 7% skimmed milk, the blots were incubated overnight at 4°C with primary antibodies against JEV E-glycoprotein (Abcam, USA), and JEV NS5 (a kind gift from Dr. Chun-Jung Chen, Taichung Veterans General Hospital, Taichung, Taiwan), iNOS (Upstate-Chemicon, USA), HSP-70, SOD-1 (Santa Cruz Biotechnology, CA, USA), TRX (AB Frontiers, Korea; a kind gift from Dr. Ellora Sen, NBRC), phospho NFκB, phospho ERK1/2, total ERK1/2 and phosphoP38 MAP kinase (Cell Signaling, USA) at 1∶1000 dilutions. After extensive washes with PBS–Tween, blots were incubated with appropriate secondary antibodies conjugated with peroxidase (Vector Laboratories, CA, USA). The blots were again washed with PBS–Tween and processed for development using chemiluminescence reagent (Millipore, USA). The images were captured and analyzed using Chemigenius, Bioimaging System (Syngene, Cambridge, UK). The blots were stripped and reprobed with anti-β-tubulin (Santa Cruz Biotechnology, USA) to determine equivalent loading of samples [22].

### Immunohistochemistry

For immunohistochemical staining, brains from scarified animals were excised following repeated transcardial perfusion with ice-cold saline and fixed with 4% paraformaldehyde. Twenty micron thick cryosections were made with the help of Leica CM3050S cryostat and processed for immunohistochemical staining to detect presence of JEV antigen in the brain and to label activated microglia. Sections were incubated overnight at 4°C with mouse anti-JEV antigen (Nakayama, 1∶250) (Chemicon, CA, USA) and rabbit anti-Iba-1 (1∶ 500; Wako, Osaka, Japan), respectively. After washes, slides were incubated with FITC-conjugated anti-mouse or anti-rabbit secondary antibodies (Vector laboratories Inc. Burlingame, USA) and following final washes, sections were sections were cover slipped after mounting with 4′-6-diamidino-2-phenylindole (DAPI, Vector laboratories Inc.). The slides were observed under Zeiss Axioplan 2 fluorescence microscope and Zeiss Apotome microscope (Zeiss, Gottingen, Germany), respectively [21].

### Thionin staining

Cryosections of brain from Sham-treated, JEV-infected and JEV-infected and MO treated animals were rinsed in de-ionized water followed by incubation with the thionin dye. The excess dye was washed off and the slides were immersed in alcohol-dioxane (1∶1) solution for differentiation. After two changes in xylene the slides were mounted with DPX and observed under a Leica 4000 DB light microscope (Leica Microsystems, USA) [23].

### Reactive oxygen species (ROS) assay

The level of ROS produced within brain tissue of each treatment groups were measured by the cell permeable, non-polar, H_2_O_2_-sensitive probe 5(and 6)-chlromethyl-20,70-dichlorodihydrofluoresceindiacetate (CM-H2DCFDA; Sigma, USA). CM-H2DCFDA diffuses into cells, where its acetate groups are cleaved by intracellular esterases, releasing the corresponding dichlorodihydrofluorescein derivative. Subsequent oxidations of CM-H2DCFDA yields a fluorescent adduct dichlorofluorescein that is trapped inside the cell. Brain homogenates were treated with 5 µM solution of CM-H2DCFDA followed by incubation in dark at room temperature for 45 min and then the relative fluorescence intensity were measured with the help of Varioskan Flash multimode reader (Thermo Electron, Finland) at excitation 500 nm and emission 530 nm. The fluorescence intensity of intracellular CM-H2DCFDA is a linear indicator of the amount of H_2_O_2_ in the cells. The measured mean fluorescence intensity was then normalized to equal concentrations of protein in each sample [23].

### Nitric oxide (NO) assay

Nitric oxide released from brain homogenates following MO treatment was assessed using Griess reagent as described previously. Briefly, 100 µL of Griess reagent (Sigma, St. Louis, USA) was added to 100 µL of brain homogenate and incubated in dark for 15 min. The intensity of the color developed was estimated at 540 nm with the help of a Benchmark plus 96-well ELISA plate reader (Biorad, CA, USA). The amount of nitrite accumulated was calculated (in µM) from a standard curve constructed with different concentrations of sodium nitrite [21].

### Intracellular staining by flow cytometry for JEV antigen

Mouse neuroblastoma cells (N2a) were plated in five 60 mm plates at a density of 5×10^5^ cells/plate, and were cultured for 18 h. After 6 h in serum free DMEM, cells were either mock-infected with sterile 1× PBS or infected with JEV at multiplicity of infection (MOI) of 5. After 1½ h, cells were washed twice with sterile 1× PBS to remove non-internalized virus. Three of the four plates that were infected with JEV, were treated with SC-MO, 3′ MO and 5′ MO at 10 µM concentrations and all plates were incubated for 24 h in serum free media.

After two washes with 1× PBS, cells were first fixed with BD cytofix solution (BD Biosciences) for 15 min and permeabilized by resuspending in permeabilization buffer (BD Cytoperm plus; BD Biosciences) and incubated at 25°C for at least 10 min. Cells were then washed twice in wash buffer (PBS containing 1% bovine serum albumin) then resuspended in wash buffer at 1×10^6^ cells per 100 µL. Primary antibody (JEV Nakayama strain; Chemicon, USA) were added in 1∶100 dilutions and incubated for 30 min at 25°C. The cells were washed with wash buffer and pelleted by centrifugation followed by incubation with FITC conjugated secondary antibody for 30 min. After final wash with wash buffer, cells were resuspended in 400 µL FACS buffer and analyzed on a FACS Calibur. The percentage of population of JEV-positive cells was calculated after gating the populations on a Dot plot using Cell Quest Pro Software (BD Biosciences).

### Statistical analysis

Statistical analysis was performed using SIGMASTAT software (SPSS Inc., Chicago, IL, USA). Data were compared between groups using one-way analysis of variance followed by post hoc test. Differences upto p<0.05 were considered significant.

## Results

### MOs confer protection to animal from Japanese encephalitis

**Figure 2:**
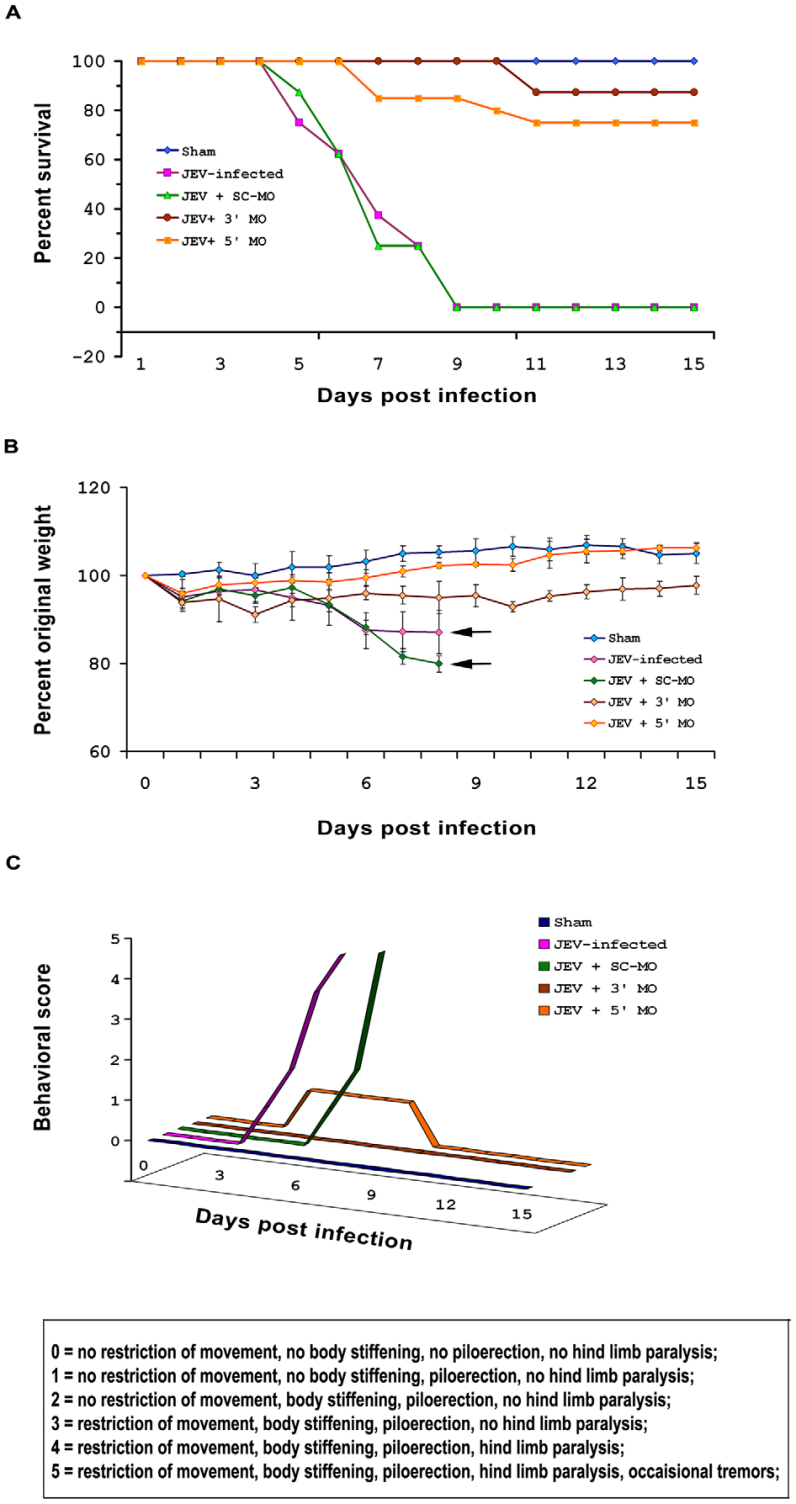
Mice are protected from JEV following MO treatment. The survival of mice following JEV infection was dramatically increased with treatments of both 3′ and 5′ MO, though the survival in 3′ MO treated mice was greater (∼90%) than those treated with 5′MO (75%) (A). Considerable changes in the average body weights of JEV-infected animals treated with both 3′ and 5′ MO were not observed when compared to animals belonging to JEV and JEV+ SC-MO groups that showed significant reductions in their body weights from 6^th^ day post infection till their death. Black arrows points to the days by which all the animals died. (B). Infection with JEV was accompanied with distinct symptoms that were alleviated following treatments with both 3′ and 5′ MO. Animals were assigned scores according to the symptoms, in a blinded manner. The graph was plotted by taking the scores of one animal that was considered as the representative of that group (C). n = 8 for all experiments; data shown are representative of duplicate sets of experiments.

MO treatment conferred significant protection to mice following JEV infection. The survival of mice following JEV infection was dramatically increased with treatments of both 3′ and 5′ MO. Approximately 90% of all the animals that were treated with 3′ MO survived as compared to 75% survival of those animals that were treated with 5′MO, post infection with JEV *(* *Figure 2A* *)*. Infection with JEV was accompanied with distinct symptoms and weight loss whereas treatments with both 3′ and 5′ MO post JEV infection, prevented animals from suffering. Not much considerable changes in the average body weights of JEV-infected animals treated with both 3′ and 5′ MO were observed when compared to animals belonging to JEV and JEV+ SC-MO groups showing significant reductions in their body weights 6 days post infection *(* *Figure 2B* *)*. The symptoms associated with JE in murine model were observed on daily basis and scores were attributed accordingly. The animals that had most symptoms received the highest scores. It was observed that 3′ and 5′ MO treated animals scored lesser than those belonging to the JEV-infected or JEV+SC-MO groups *(* *Figure 2C* *)*.

### Reduction of viral load *in vivo* due to MO treatment

**Figure 3:**
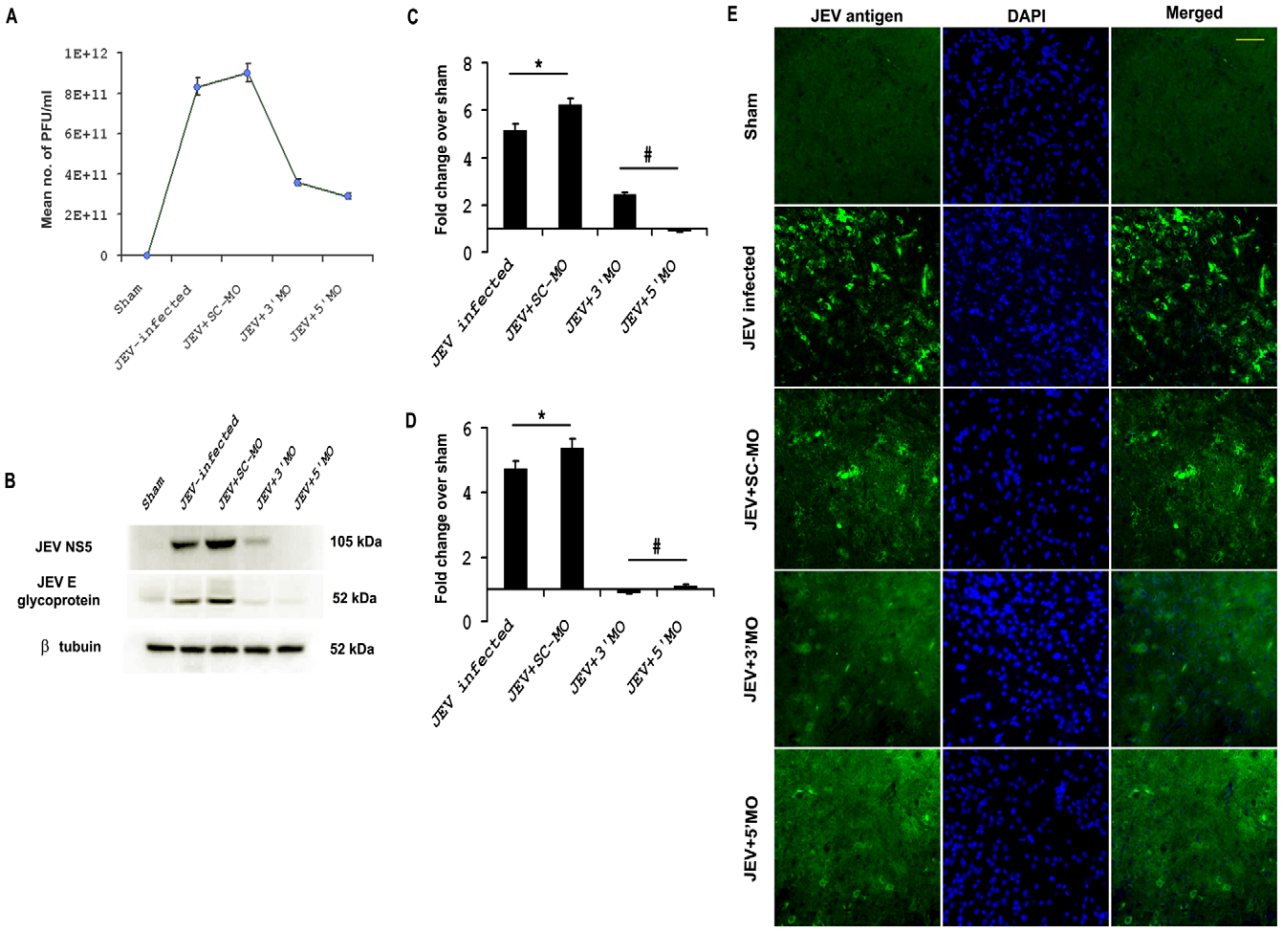
MOs treatment reduces the viral load *in vivo*. Significant reduction in viral titer was observed following MO treatment in animals, as compared to JEV-infected and JEV+SC-MO groups. (^*^ p<0.001 for JEV and JEV+SC-MO when compared to Sham, ^#^ p<0.001 for JEV+ 3′MO and JEV+ 5′ MO when compared to only JEV-infected group) (*A*). Immunoblot analysis showed expression of JEV NS5 and E glycoprotein were significantly increased in JEV-infected and JEV+SC-MO than Sham, but reduced significantly after both 3′ and 5′ MO treatments (^*^ p<0.01 for JEV and JEV+SC-MO when compared to Sham, ^#^ p<0.01 for JEV+ 3′MO and JEV+ 5′ MO when compared to only JEV-infected group) (*B–D*). Immunostaining of brain sections from different treatment groups showed greater presence of JEV antigen in JEV and JEV+SC-MO groups (*E*). Magnification ×20; scale bar correspond to 50µ Photomicrographs shown here in this figure are representative of three individual animals from each group.

To assess whether the MOs has any effect on reduction of viral load in brain, homogenized brain samples from all the treatment groups were subjected to plaque assay as described in materials and methods section. Number of PFU/mL of the brain homogenate was found to be significantly higher in both JEV and JEV+SC-MO groups when compared to Sham (p<0.001). Viral PFUs were found to be significantly reduced following 3′ MO and 5′ MO treatment when compared to only JEV-infected or JEV+SC-MO group (p<0.001) *(* *Figure 3A* *)*.

To further validate the results obtained from the plaque assay, immunoblot for some of the JEV-specific proteins were performed. The expression of NS5, a non structural protein of JEV, was significantly increased in JEV and JEV+SC-MO groups when compared to Sham (p<0.01), but its level were found to be significantly reduced after both 3′ and 5′ MO treatments when compared to JEV-infected group (p<0.01). Similarly, E glycoprotein level showed significant increase in JEV-infected and JEV+SC-MO groups when compared to Sham (p<0.01) which were then drastically reduced following 3′ and 5′ MO treatments (p<0.01) *(* *Figure 3B–D* *)*. Immunostaining of brain sections showed greater presence of JEV antigen in JEV-infected and JEV+SC-MO groups, whereas 3′ and 5′ MO treatments resulted in lesser presence *(* *Figure 3E* *)*.

### MOs abrogates neurodegeneration, microglial activation and release of proinflammatory cytokines in the brain

**Figure 4:**
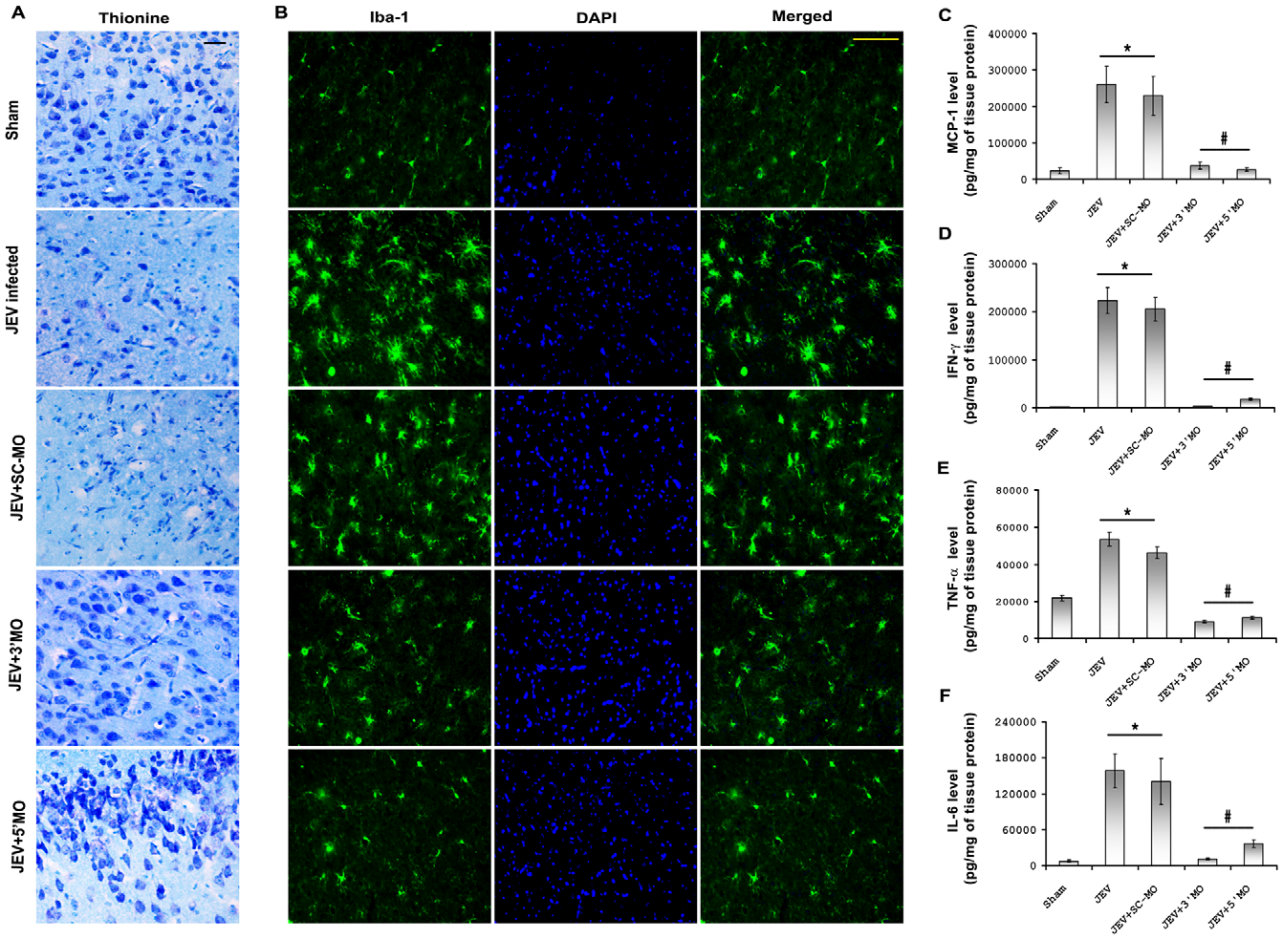
MOs neuroprotect, reduces microglial activation and inhibits proinflammatory cytokines production in brain. Thionin staining of brain sections from all the treatment groups showed neurons with distinct morphology in Sham-treated, JEV+3′ MO and JEV+5′ MO groups. However in sections from JEV-infected and JEV+SC-MO groups showed damaged neurons with altered morphology. Magnification ×20; scale bar correspond to 50 µ. Immunofluorescent staining for microglia-specific Iba-1 performed in brain sections of all groups showed that number of activated (star shaped) microglia appeared to be more frequent in JEV-infected and JEV+SC-MO groups as compared to compared to sections belonging to Sham, JEV+3′ MO and JEV+5′ MO groups. Magnification ×20; scale bar correspond to 50 µ (B). Photomicrographs shown here in this figure are representative of three individual animals from each group. CBA showed levels of MCP-1, IFN-γ, TNF-α, and IL-6 were increased significantly in both JEV-infected and JEV+SC-MO groups when compared to Sham treated groups. The elevated levels of these proinflammatory cytokines were then significantly reduced with 3′ and 5′ MO treatments (^*^ p<0.01 for JEV and JEV+SC-MO when compared to Sham; ^#^ p<0.01 for JEV+ 3′MO and JEV+ 5′ MO when compared to only JEV-infected group) (C–F).

To further characterize the inhibitory effects of MO on JEV-induced neuronal death, brain sections from all the treatment groups were subjected to thionin staining. Numerous healthy cells were seen in sections obtained from Sham, JEV+3′ MO and JEV+5′ MO groups when compared to sections belonging to only JEV-infected or JEV+SC-MO groups which contained numerous unhealthy/dying neurons with altered morphology *(* *Figure 4A* *)*.

Microglial activation and increased proinflammatory cytokine production are the hallmarks of JEV infection [24]. To see whether MO treatment helps in vitiation of these effects, immunostaining for microglial specific marker Iba-1 was performed in brain sections of all treatment groups. In brain sections of JEV and JEV+SC-MO groups the number of activated microglia with characteristic morphology, appeared to be more frequent when compared to sections belonging to Sham, JEV+3′ MO and JEV+5′ MO groups *(* *Figure 4B* *)*. CBA performed to check the proinflammatory cytokines levels in the brain homogenates obtained from different treatments showed that levels of MCP-1, IFN-γ, TNF-α, and IL-6 were found to be significantly increased in both JEV and JEV+SC-MO groups when compared to Sham infected groups (p<0.01). The elevated levels of these proinflammatory cytokines were drastically reduced with 3′ and 5′ MO treatments (p<0.01) *(* *Figure 4C–F* *).*

### Modulations of oxidative stress in brain following MO treatment

**Figure 5:**
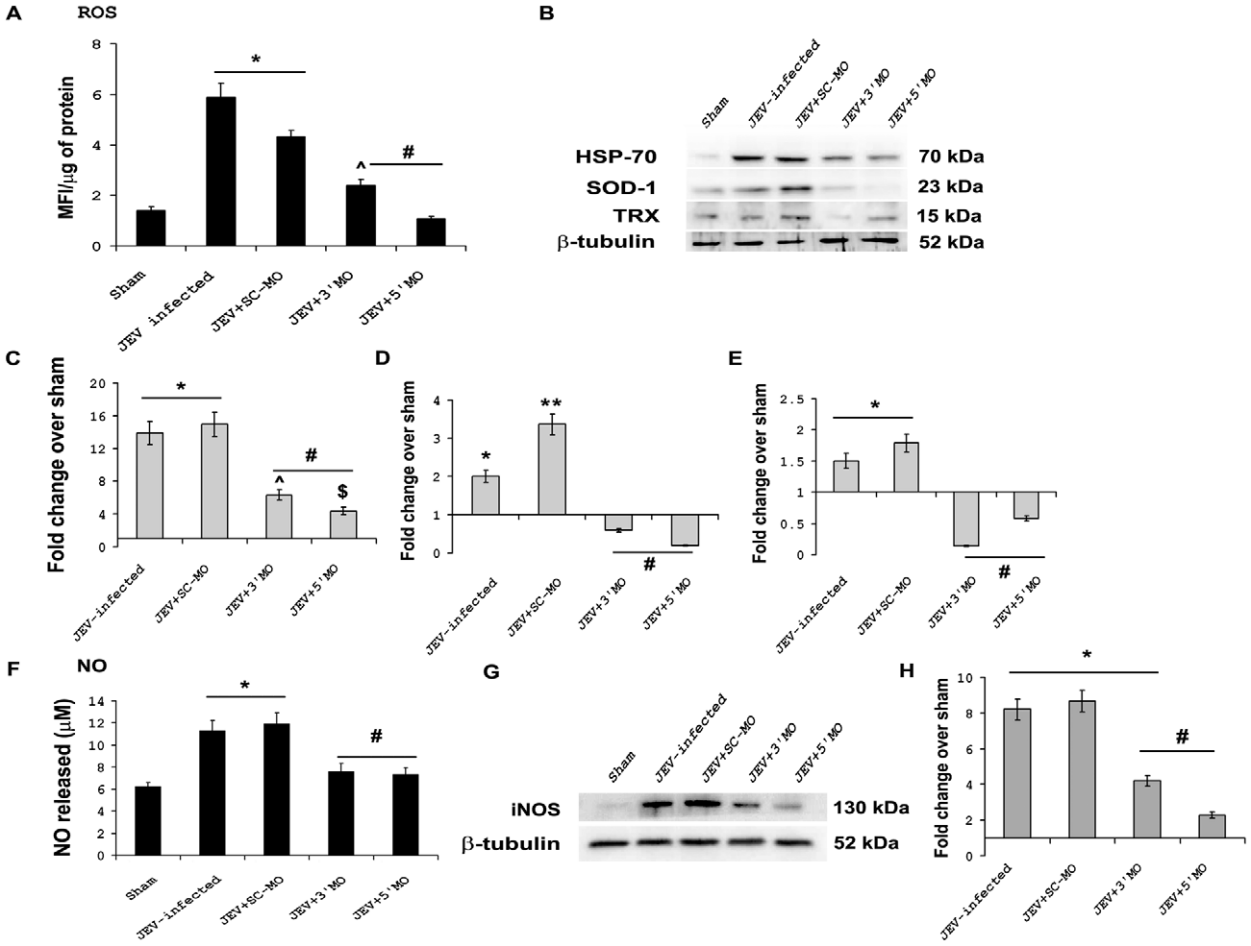
MOs treatment modulates oxidative stress markers in CNS. Increases were observed in the ROS levels in the brain samples of JEV-infected and JEV+SC-MO groups in comparison to Sham, that were reduced following 3′ and 5′ MO treatments. Although ROS levels were decreased in JEV+3′ MO group when compared to JEV-infected group, it remained significantly higher than that in Sham (A). Approximately 13-fold increases were observed in the levels of HSP-70 in JEV-infected and JEV+SC-MO groups as compared to Sham. These drastic increases were significantly reduced in JEV+3′ MO and JEV+5′ MO groups, the levels remained significantly higher than Sham (B&C). SOD-1 levels were found to be elevated 2- and nearly 3-fold in JEV-infected and JEV+SC-MO groups respectively compared to Sham. 3′ and 5′ MO treatment caused significant reduction of SOD-1 levels compared to JEV-infected group (B&D). Alterations in TRX-1 levels were similar to that observed in SOD-1 except that its level in JEV+ SC-MO was not significantly different than only JEV-infected group (B&E). Nearly 2-fold increases were observed in NO levels of JEV-infected and JEV+SC-MO groups when compared to those obtained from Sham. NO levels were subsequently reduced to significantly lower levels following 3′ and 5′MO treatments (F). iNOS expression was found to increase 8-fold in JEV-infected and JEV+SC-MO groups when compared to Sham. Following 3′ and 5′MO treatments iNOS levels decreased significantly as compared to JEV-infected group (G&H). (^*^ p<0.01 for JEV when compared to Sham; ^**^ p<0.01 for JEV+SC-MO when compared to JEV-infected only;^ #^ p<0.01 for JEV+ 3′MO and JEV+ 5′ MO when compared to only JEV-infected group; ^ p<0.01 for JEV+ 3′MO when compared to Sham; ^$^ p<0.01 for JEV+ 5′ MO when compared to Sham).

Increased oxidative stress in CNS is a major outcome of JEV infection [20]. To evaluate whether MO treatment of mice resulted in abrogation of oxidative stress following JEV infection, we measured ROS and NO levels in brain homogenate obtained from all treatment groups. Two fold increases were observed in the ROS levels in the brain samples of JEV and JEV+SC-MO groups when compared to Sham (p<0.01), significant reduction in the ROS levels were observed in JEV+3′ MO and JEV+5′ MO groups when compared to only JEV-infected groups (p<0.01). Although ROS levels has decreased in JEV+3′ MO group when compared to JEV group, it remained significantly higher than that of Sham (p<0.01) *(* *Figure 5A* *).* Superoxide dismutase 1 (SOD-1) and Thioredoxin (TRX-1) are the proteins associated with oxidative stress. SOD-1 levels were found to be elevated approximately 2- and 3-fold in JEV-infected and JEV+SC-MO groups respectively when compared to Sham (p<0.01). Its levels in JEV+3′ MO and JEV+5′ MO groups were reduced significantly when compared to JEV-infected group (p<0.01). TRX-1 levels were also found to be increased significantly in JEV-infected and JEV+SC-MO groups when compared to Sham (p<0.01) but it were significantly reduced in brain samples obtained from JEV+3′ MO and JEV+5′ MO groups when compared to only JEV-infected groups (p<0.01) *(* *Figure 5B, D&E* *).* HSP-70 is a heat shock protein that has been associated with intracellular stress. Significant twelve fold increases in the levels of HSP-70 were observed in JEV and JEV+SC-MO groups when compared to Sham (p<0.01), this drastic increases in the levels of HSP-70 in JEV and JEV+SC-MO groups were reduced in JEV+3′ MO and JEV+5′ MO groups (p<0.01) *(* *Figure 5B&C* *).*

JEV infection leads to increased nitric oxide (NO) production in CNS [25]. Significant two fold increases were seen in the NO levels in brain samples obtained from JEV-infected and JEV+SC-MO groups when compared to those obtained from Sham (p<0.01). NO levels subsequently got down to significantly lower levels following 3′ and 5′MO treatments (p<0.01) *(* *Figure 5F* *).* Immunoblot analysis showed nearly 8-fold increases in levels of iNOS in JEV-infected and JEV+SC-MO groups when compared to Sham (p<0.01). iNOS levels showed significant decreases in JEV+3′ MO and JEV+5′ MO groups when compared to only JEV-infected groups (p<0.01) *(* *Figure 5G&H* *).*

### MO treatment modulates the expression pattern of key proteins associated with stress

**Figure 6:**
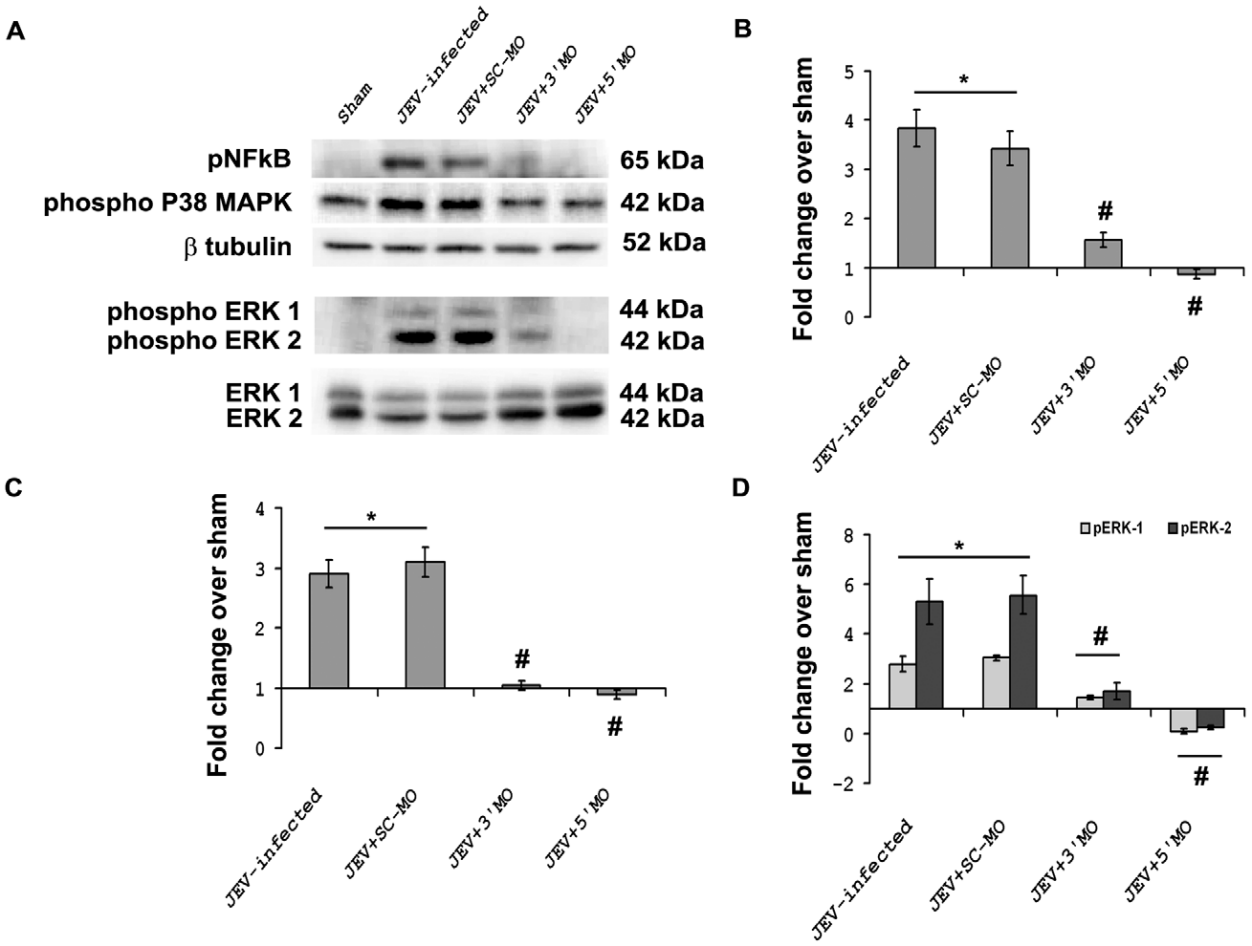
MOs regulates the expression pattern of several key proteins associated with stress. Approximately 4-fold increases in the levels of pNFκB and 3-fold increases in the levels of phosphoP38MAPK in JEV-infected and JEV+SC-MO groups were observed in comparison to Sham. The levels of both pNFκB and phosphoP38MAPK were significantly reduced in JEV+3′ MO and JEV+5′ MO groups when compared to only JEV-infected groups (A–C). Phospho ERK1 and ERK2 levels were found to be increased by approximately 3- and 5-fold in JEV-infected and JEV+SC-MO groups respectively, when compared to Sham. but showed considerable decreases in JEV+3′ MO and JEV+5′ MO groups when compared to only JEV-infected groups (A&D) (^*^ p<0.01 for JEV-infected and JEV+SC-MO when compared to Sham; ^#^ p<0.01 for JEV+3′ MO and JEV+5′MO when compared to only JEV-infected groups).

Western blot analysis demonstrated a significant inhibition in the expression of different stress related proteins whose levels were elevated following JEV infection. Upon MO treatments there were approximately 4-fold increases in the levels of pNFκB in JEV and JEV+SC-MO groups when compared to Sham (p<0.01). The levels of pNFκB were found to be significantly reduced in JEV+3′ MO and JEV+5′ MO groups when compared to only JEV-infected groups (p<0.01) *(* *Figure 6A&B* *).* Phospho p38 MAPK levels also showed significant 3-fold increases in JEV and JEV+SC-MO groups when compared to Sham (p<0.01), its levels were also found to be reduced significantly following treatment with 3′ and 5′ MO when compared to only JEV-infected groups (p<0.01) *(* *Figure 6A&C* *).* Both phospho ERK1 and ERK2 levels were found to be significantly increased in JEV and JEV+SC-MO groups when compared to Sham (p<0.01). The levels of phospho ERK1 and ERK2 showed considerable decreases in JEV+3′ MO and JEV+5′ MO groups when compared to only JEV-infected groups (p<0.01) *(* *Figure 6A&D* *).*

### MO treatment reduced viral titer *in vitro* and decreased the intracellular viral load

To assess whether MO has any effect on viral load *in vitro* N2a cell lysates from all the treatment groups were subjected to plaque assay. PFU/mL of the cell lysates was found to be significantly higher in both JEV and JEV+SC-MO groups when compared to mock-infected cells (p<0.01). Viral loads were found to significantly reduced in both JEV+3′ MO and JEV+5′ MO groups when compared to only JEV-infected group (p<0.01) *(Figure S1A)*. To further ascertain the results obtained from plaque assay, intracellular staining of JEV antigen in N2a was performed and number of JEV-positive N2a cells was then counted by flow cytometry. Only 16% and 9% of the total gated cells were found to be positive for JEV antigen in JEV+3′ MO and JEV+5′ MO groups respectively as compared to 30% in JEV-infected group, and 34% in JEV+SC-MO group *(Figure S1B)*.

## Discussion

Use of anti-sense molecules for targeted inhibition of viral replication has been under investigation for quite sometime. Though the application of these molecules has raised the possibilities of their future use as novel therapeutic agents, there are many issues regarding their effectiveness in terms of their stability and delivery to targeted cells. Recent studies are involved in developing techniques to minimize or eliminate these issues so that anti-sense therapy can be employed to a wide variety of intractable diseases such as splice-modifying genetic defects and viral diseases. The role of various anti-sense molecules in the inhibition of replication of JEV has been reported with positive outcomes [10], [11], [12], [13], [26].

Morpholino oligomers are single stranded anti-sense molecules that exert their action by steric blocking of complementary RNA. Unlike other types of anti-sense oligonucleotides, Morpholinos provide all the desired properties of stability, nuclease resistance, high efficacy, long-term activity, water solubility, low toxicity, and exquisite specificity. Morpholino oligomers has been used previously for the inhibition of flaviviral replication [14], [27] including JEV [15] though all of them has utilized peptide based Morpholinos. The peptide based Morpholinos contain delivery moiety evolved from natural peptides whose active components are 6–9 arginine residues in a bio-available 6-aminohexanoic-spaced structure [28]. However, these arginine-based peptides are not commercially available for research purposes and their greatest efficacies have been in delivering Morpholinos to the cytosol of tissues like liver [29] or leaky muscle [30], which would be considered as easily deliverable. As a result, the reach of peptide based Morpholinos into a wide spectrum of tissues remains questionable [18]. Also, owing to the peptidic nature, degradation of the peptide portion of the conjugates was found to be time and tissue dependent [31]. Furthermore, the applications of the arginine-rich peptide transporters are limited due to their high cost, scalability and stability. Added to that are the risks of immune responses against the peptides which limits repeated administrations for diseases requiring long-term treatment [32].

To minimize the problems encountered by the peptide-conjugated Morpholinos, octaguanidinium dendrimer-conjugated Morpholino oligomers have been developed that are commonly referred to as Vivo-Morpholino (MO). These custom-sequence anti-sense molecules have been reported to enable Morpholino applications in adult animals. MO was our choice of anti-sense molecule as this enabled us to test the specifically designed oligonucleotides in both animal as well as cell culture models. Though ‘outstanding’ results have been reported to be achieved by intravenous (i.v.) administration of the MO, we preferred the intraperitoneal route via which modest systemic delivery can be achieved. This was so done because brain has been reported to be an ineffective tissue when MOs are administered i.v. [33], though there is no direct evidence showing that MOs can cross blood brain barrier, when administered via other routes. According to the manufacturer's (Gene Tools LLC) instructions the maximum suggested dosage in mammals is 12.5 mg/kg in a 24 hour period. Our aim was to determine the minimum dose at which our desired effects could be achieved. Initially we had chosen two doses of 5 mg and 10 mg per kg body weight (b.w.) of the animals. We found that there was no significant difference between the survival rate of JEV-infected and MO treated mice in either dose *(data not shown for 10 mg/kg b.w.)*. The survival rate was approximately 90% in those JEV-infected animals that were treated with 3′ MO and 75% in animals treated with 5′ MO. Thus we decided to proceed with the 5 mg/kg b.w. dose for all subsequent experiments.

Plaque assay from the brain homogenates of animals of all groups revealed that the number of infective viral particle production was dramatically reduced following 3′ and 5′ MO treatment. The 3′ MO was generated against the 3′ CSI region of the JEV genome that interacts with 5′ CS region located in coding sequence for capsid protein at 136–146 nucleotides from 5′ terminal of the genome. This interaction results in cyclization of JEV genome that is necessary for its efficient replication. The 5′ MO was targeted towards one of the secondary structures of the 5′ UTR that are required for the formation of translation pre-initiation complex. Blocking of these two sites in the JEV genome leads to the most likely effect, i.e. inhibition of replication and translation of viral genome. This was further corroborated by the decrease in the expressions of viral proteins (NS5, E glycoprotein and general flaviviral envelop protein) in the brain. Flaviviral NS5 is known to possess guanylyltransferase activity that helps in the synthesis of methylated cap structure at the 5′ end of the viral genome that plays a crucial role in the translation and stability of mRNAs [34]. The JEV E glycoprotein is believed to be involved in viral adhesion and entry into host cells, hemagglutination, cellular tropism, viral virulence, and the induction of protective immune responses [35]. Decreased expression of these proteins indicates that viral replication and production of new infective viral particles are inhibited due to the MOs. Immunohistochemical staining for viral antigen also provided visual confirmation of the fact that JEV antigen was detected at much lower amounts in the brain following MO treatment. However, these data does not prove that MOs directly inhibit infective viral particle production in the brain itself, as it cannot be conclusively stated whether the MOs can reach brain. These data merely suggests that the number of replication-competent infective JEV in the brain was significantly reduced, which subsequently leads to neuroprotection.

It is well known that JEV infection causes microglial activation. Activated microglia releases an array of chemical mediators that are detrimental for the neurons in brain [24]. Since there was reduction in the production of infective viral particles following 3′ and 5′ MO treatments, we studied the effect on microglial pathophysiology in mouse brain. Our results show that there was significantly reduced number of activated microglia in the brain sections of both 3′ and 5′ MO-treated animals as compared to only JEV-infected or JEV-infected and SC-MO treated animals. Since there were little or no activation of microglia, proinflammatory cytokine levels in the brain were found to be significantly downregulated. Histochemical staining also revealed that neuronal population and morphology remained largely unaffected in 3′ and 5′ MO-treated animals' brains as compared to only JEV-infected or JEV-infected and SC-MO treated animals.

Generation of ROS with the generation of oxidative damage has been implicated in neurodegenerative diseases and in the degradation of nervous system functions and are also reported to increase following JEV infection [22]. Increase in ROS levels initiates various responses within the cell, including damage to proteins, DNA and lipid [36]. In this study, ROS levels were found to be many-fold increased in JEV-infected or JEV-infected and SC-MO treated animals that were then found to be counteracted by the treatment of 3′ and 5′ MO. The levels of stress related proteins such as SOD-1, HSP-70 and TRX where also found to be positively modulated following 3′ and 5′ MO treatment. NO is a known antagonist of JEV. It has been shown that NO inhibits JEV infection by preventing viral replication [37]. In our study NO levels were increased in the brain in response to JEV infection possibly due to the upregulation of inducible nitric oxide synthase (iNOS). Treatments with 3′ and 5′ MO caused a decrease of NO to basal levels as observed in Sham-treated animals.

Activation of pNFκB regulates apoptotic genes, especially the TRAF1 and TRAF2, and thereby checks the activities of the caspases, which are central to most apoptotic processes. JEV is known to activate pNFκβ via a PI3K-dependent pathway in the brain of infected animals, which is associated with apoptosis [38]. JEV infection has also been shown to activate stress kinases, which in turn results in activation of ERK1/2, and p38 MAPK pathway leading to apoptotic death of neurons [39]. In accordance with the established results, here also we found that there was similar activation pattern of these molecules in JEV-infected and JEV-infected and SC-MO treated animal brain samples. Treatment with the MOs resulted in abrogation of those changes that led to greater survivality of brain neurons as observed by histochemical staining. Activation of p38MAPK is also related to the transcriptional activation of proinflammatory genes in the brain [40]. Thus the decrease in phophoP38 MAPK levels correlates with the decreased levels of proinflammatory cytokine levels in obtained from the brain.

To confirm the anti-viral and neuroprotective property of the MOs observed in *in vivo* models, cultured neuroblastoma cells were infected with JEV, followed by MO treatment. Though the MOs are specifically developed for *in vivo* studies, they are also known to be taken up by cells in culture conditions [18]. There was a significant decrease in viral titer in samples obtained from the cells that were treated with 3′ and 5′ MOs as compared to either JEV-infected or JEV-infected and SC-MO treated cells, as revealed by plaque assay. This data was supported by FACS analysis following intracellular staining for JEV antigen.

This study was undertaken to determine the antiviral and neuroprotective efficacy of Vivo-Morpholinos in an experimental model of JE so that it can be considered as a therapeutic agent in the near future. There have been studies regarding the anti-JEV effects of other types of Morpholino oligomers though none of them are yet to be considered for therapeutic purposes. This is the first study that investigates the role of Morpholino oligomers specially designed for effective delivery into live animal models. Generally, the i.p route of administration of any drug is preferred in animal studies over any other routes. However, the efficacy of these antisense molecules needs to be checked by administering through other applicable routes, as i.p. administration in humans is uncommon, though not unheard of. The amounts of oligomers required and the route of administration in this study marks these molecules as practicable therapeutic agents in JE, though further studies are required before these can be recommended for clinical trials.

## Supporting Information

Figure S1 MO treatments decrease viral load *in vitro*. Mouse neuroblastoma cell (N2a) lysates from all the treatment groups were subjected to plaque assay in order to determine viral loads. PFU/mL was found to be significantly higher in both JEV and JEV+SC-MO groups when compared to Sham. Viral loads were found to be significantly reduced in both JEV+3′ MO and JEV+5′ MO groups when compared to only JEV-infected group (* p < 0.01 for JEV and JEV+SC-MO when compared to Sham; # p < 0.01 for JEV+3′ MO and JEV+5′MO when compared to only JEV-infected group) (A). Intracellular staining for JEV antigen in N2a was performed and number of JEV-positive N2a cells was then sorted by flow cytometry. 30% of the total gated cells were found to be positive for JEV antigen in JEV-infected group as compared to 34% in JEV+SC-MO group. Only 16% and 9% of the total gated cells were found to be positive for JEV antigen in JEV+3′ MO and JEV+5′ MO groups respectively (B).
(0.25 MB TIF)
