# Supplementary figures and images for: Antiviral and Neuroprotective Role of Octaguanidinium Dendrimer-Conjugated Morpholino Oligomers in Japanese Encephalitis
Source: PLoS Negl Trop Dis. 2010 Nov 23;4(11):e892. doi: 10.1371/journal.pntd.0000892 (PMC2990691; doi:10.1371/journal.pntd.0000892)

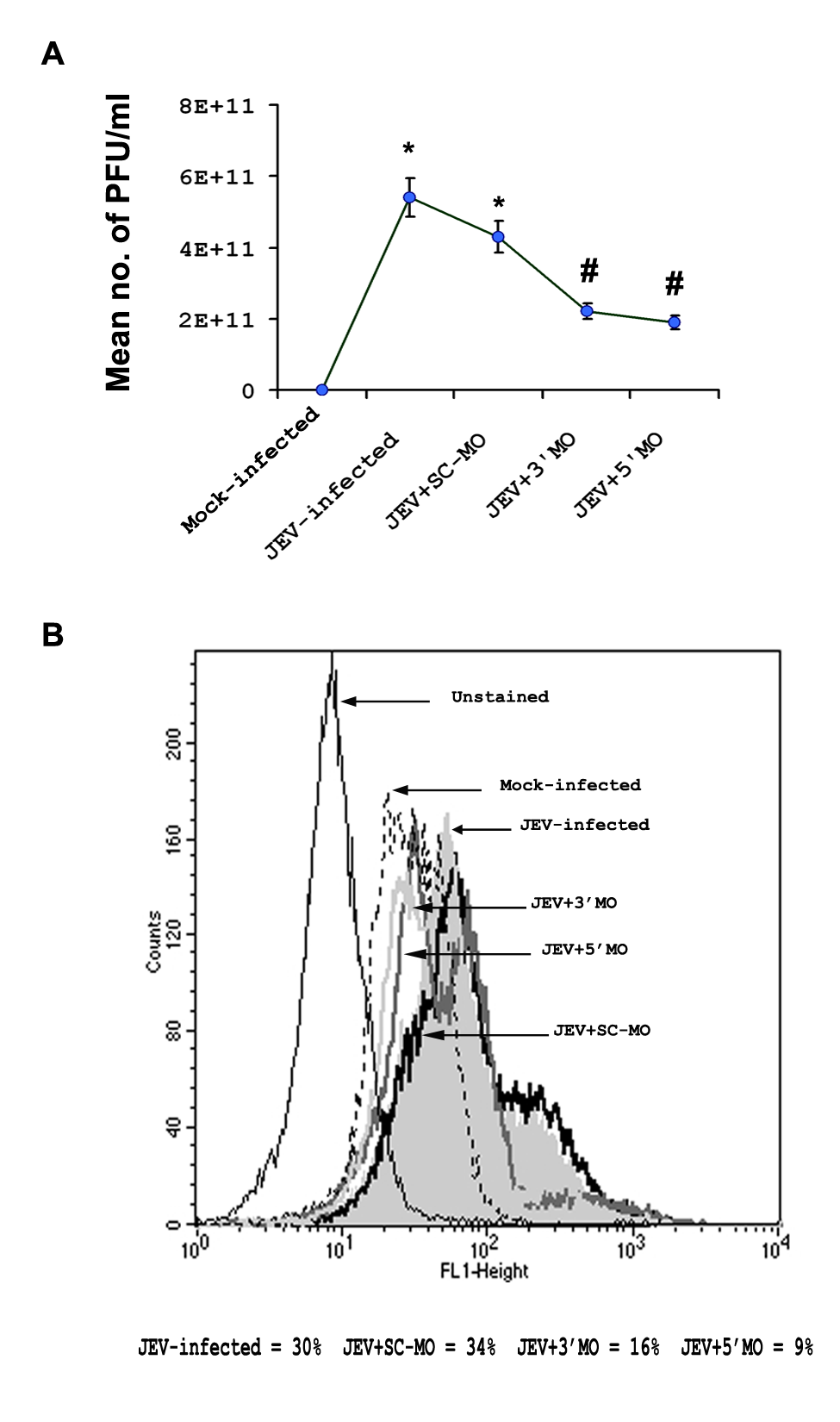

Supplement: Figure S1 — MO treatments decrease viral load in vitro. Mouse neuroblastoma cell (N2a) lysates from all the treatment groups were subjected to plaque assay in order to determine viral loads. PFU/mL was found to be significantly higher in both JEV and JEV+SC-MO groups when compared to Sham. Viral loads were found to be significantly reduced in both JEV+3′ MO and JEV+5′ MO groups when compared to only JEV-infected group (* p < 0.01 for JEV and JEV+SC-MO when compared to Sham; # p < 0.01 for JEV+3′ MO and JEV+5′MO when compared to only JEV-infected group) (A). Intracellular staining for JEV antigen in N2a was performed and number of JEV-positive N2a cells was then sorted by flow cytometry. 30% of the total gated cells were found to be positive for JEV antigen in JEV-infected group as compared to 34% in JEV+SC-MO group. Only 16% and 9% of the total gated cells were found to be positive for JEV antigen in JEV+3′ MO and JEV+5′ MO groups respectively (B). (0.25 MB TIF) [file pntd.0000892.s001.tif]
